# Selective Postoperative Hemoglobin Testing After Cesarean Delivery: A Predictive Model for Severe Postpartum Anemia

**DOI:** 10.7759/cureus.106109

**Published:** 2026-03-30

**Authors:** Abbey P Donahue, Jeff Laux, Aileen J Beckham, James Edwards

**Affiliations:** 1 Obstetrics and Gynecology, Atrium Health Wake Forest Baptist, Winston-Salem, USA; 2 Biostatistics, University of North Carolina at Chapel Hill, Chapel Hill, USA; 3 Obstetrics and Gynecology, WakeMed Hospitals, Raleigh, USA

**Keywords:** cesarean delivery, elastic net regression, hemoglobin testing, obstetric hemorrhage, postpartum anemia, postpartum transfusion, predictive model, quality improvement, risk stratification

## Abstract

Introduction: Cesarean delivery is one of the most common surgeries in the United States and is associated with greater postpartum blood loss than vaginal delivery. Despite increasing emphasis on selective, symptom-driven evaluation, routine postoperative hemoglobin testing following cesarean delivery remains common practice. Identifying patients at greatest risk for severe postpartum anemia may help guide more targeted laboratory assessment while avoiding unnecessary testing in low-risk individuals. This study aimed to develop and evaluate a predictive model for severe postpartum anemia following cesarean delivery.

Methods: We conducted a retrospective cohort study of patients undergoing cesarean delivery at three hospitals within the WakeMed Health and Hospitals system in North Carolina during 2019. Patients aged ≥18 years with both preoperative and postoperative hemoglobin measurements were included. Demographic, obstetric, and clinical variables were extracted from the electronic health record. Severe postpartum anemia was defined as postoperative hemoglobin <7 g/dL. Elastic net logistic regression with 10-fold cross-validation was used to identify predictors of severe anemia.

Results: A total of 2,061 patients met the inclusion criteria. Severe postpartum anemia occurred in 80 patients (3.8%), and 116 patients (5.6%) received blood transfusion. The final predictive model retained two variables: preoperative hemoglobin level and number of uterotonic agents administered. The model demonstrated strong discrimination for predicting severe anemia (area under the curve: 0.89). Applying a prevalence-based probability threshold of 3.5% reduced postoperative hemoglobin testing by 65% while maintaining 90% sensitivity for identifying severe anemia.

Discussion: These findings suggest that a small number of routinely available clinical factors can effectively identify patients at increased risk for severe postpartum anemia following cesarean delivery. A risk-based approach to postoperative laboratory evaluation may support current guideline recommendations favoring selective testing and may reduce unnecessary laboratory utilization without compromising patient safety.

Conclusion: A predictive model incorporating preoperative hemoglobin level and uterotonic use provides a simple method for identifying patients at the highest risk for severe postpartum anemia after cesarean delivery. Implementing selective postoperative hemoglobin testing based on risk stratification may improve resource utilization and streamline postpartum care.

## Introduction

Cesarean delivery remains one of the most commonly performed surgical procedures in the United States, accounting for approximately 32.2% of all births in 2022 [1]. Because cesarean delivery is associated with greater blood loss than vaginal delivery, postoperative hemoglobin measurement is frequently obtained to evaluate for postpartum anemia or hemorrhage-related complications [2,3]. However, routine laboratory testing following cesarean delivery remains controversial. Current professional guidance does not recommend universal postoperative complete blood count (CBC) testing; instead, organizations such as the American College of Obstetricians and Gynecologists emphasize selective, symptom-driven evaluation based on clinical indicators, including postpartum hemorrhage, tachycardia, oliguria, or symptoms of anemia [4,5]. The primary objective of this study was to develop and evaluate a predictive model to identify patients at increased risk for severe postpartum anemia following cesarean delivery, with the goal of informing a more selective, risk-based approach to postoperative hemoglobin testing.

Several studies have questioned the clinical utility of routine postoperative hemoglobin testing. In gynecologic surgery populations, routine postoperative laboratory testing rarely alters management, with most transfused patients demonstrating clinical symptoms prior to laboratory confirmation. Similar findings have been reported in obstetric populations, where routine testing following uncomplicated cesarean delivery has been shown to have limited clinical value and may increase healthcare costs without improving outcomes [4,6]. These findings have led to increasing interest in risk-based strategies to guide postoperative laboratory testing.

Despite this shift toward selective testing, practical tools to identify patients at the highest risk for clinically significant postpartum anemia remain limited. Hemoglobin levels typically decline during the early postpartum period, with the nadir occurring approximately 24-48 hours after delivery [7]. In clinical practice, however, postoperative hemoglobin measurements are commonly obtained during the first postoperative day following cesarean delivery as part of routine postoperative assessment. Identifying patients at increased risk for severe anemia may therefore help guide targeted laboratory evaluation while avoiding unnecessary testing in low-risk individuals. This approach aims to support targeted laboratory evaluation while reducing unnecessary testing in low-risk patients.

## Materials and methods

This retrospective cohort study was approved by the WakeMed Institutional Review Board (approval number: 1648913-2). Because the study used de-identified data obtained from routine clinical care, informed consent was waived in accordance with the Declaration of Helsinki.

Patients were eligible if they were ≥18 years old and underwent cesarean delivery at three hospitals within the WakeMed Health and Hospitals system in North Carolina (WakeMed Raleigh, WakeMed North, and WakeMed Cary) during the 2019 calendar year. Inclusion criteria required (1) a preoperative hemoglobin measurement obtained within 72 hours prior to cesarean delivery and (2) a postoperative hemoglobin measurement obtained within 36 hours after delivery. Intraoperative hemoglobin measurements were excluded to ensure all values reflected the true postoperative status. In contrast, exclusion criteria were age <18 years, missing preoperative or postoperative hemoglobin values, or incomplete electronic health record data required for model development.

Demographic characteristics, prenatal diagnoses (International Classification of Diseases (ICD)-10 coded), comorbidities, and intrapartum variables were abstracted from the electronic health record. Race and ethnicity were obtained from the electronic health record based on patient self-report and recorded as separate demographic variables according to standard institutional classifications. Intrapartum variables included estimated blood loss, administration of magnesium sulfate, and use of uterotonic agents, defined as misoprostol, methylergonovine, or carboprost administered within 24 hours postpartum. Oxytocin was not included because it is routinely administered following delivery. Administration of tranexamic acid (TXA) was also recorded.

Postoperative hemoglobin was defined as the lowest hemoglobin value obtained between two and 36 hours after delivery, reflecting the timeframe during which postoperative CBCs are typically obtained as part of routine postoperative care following cesarean delivery. Clinical indicators of anemia were defined a priori as tachycardia (heart rate >110 beats per minute), hypotension (systolic blood pressure ≤90 mmHg or diastolic blood pressure ≤60 mmHg), or low urine output (≤0.3 mL/kg/hr over 24 hours).

A total of 62 variables were collected for descriptive analysis. Clinically relevant predictors were selected a priori based on expert judgment and prior literature, including maternal age, race/ethnicity, parity, gestational age, delivery type, cesarean priority (scheduled vs. unscheduled), uterotonic use, estimated blood loss, preoperative hemoglobin, and maternal comorbidities.

The primary outcome was severe postpartum anemia, defined as a postoperative hemoglobin <7 g/dL. This threshold was selected because hemoglobin <7 g/dL is commonly used as a transfusion threshold for stable hospitalized patients and has been applied in prior studies evaluating severe postpartum anemia [8,9]. A secondary outcome, continuous postoperative hemoglobin level, was evaluated in an exploratory analysis.

Elastic net logistic regression was used to model the probability of postoperative hemoglobin <7 g/dL, incorporating variable selection and penalization to address multicollinearity and overfitting [10]. Ten-fold cross-validation was used to optimize model hyperparameters. Model discrimination was assessed using receiver operating characteristic (ROC) curves and area under the curve (AUC). Calibration, sensitivity, specificity, positive predictive value, negative predictive value, and confusion matrices were evaluated across clinically meaningful probability thresholds, including a prevalence-based threshold of 3.5%.

An exploratory linear regression model was also fit to identify predictors of continuous postoperative hemoglobin levels. All analyses were conducted using the R statistical software (R Foundation for Statistical Computing, Vienna, Austria). The study was not a clinical trial; therefore, no clinical trial registration number applies.

## Results

A total of 2,061 patients met the inclusion criteria. The mean maternal age was 31.1±5.5 years, and demographic characteristics are summarized in Table 1.

**Table 1:** Maternal demographic and pregnancy characteristics stratified by postoperative hemoglobin level following cesarean delivery Maternal demographic and pregnancy characteristics of patients undergoing cesarean delivery (N=2,061) stratified by postoperative hemoglobin level on postoperative day 1. Continuous variables are presented as mean (standard deviation), and categorical variables are presented as number (percentage). P-values were calculated using Student's t-test for continuous variables and chi-squared or Fisher's exact tests for categorical variables.

	Overall (N=2,061)	Hemoglobin ≥7 g/dL (N=1,970)	Hemoglobin <7 g/dL (N=80)	P-value
Mean age (years)	31.1 (5.5)	31.2 (5.4)	30.0 (6.2)	0.047
Race				
Black	561 (27.2%)	525 (26.6%)	32 (40%)	0.01
White	973 (47.2%)	943 (47.9%)	27 (33.8%)	
Asian	194 (9.4%)	188 (9.5%)	4 (5%)	
Other/unknown	333 (16.2%)	314 (15.9%)	17 (21.2%)	
Ethnicity				
Hispanic	291 (14.1%)	273 (13.9%)	16 (20%)	0.219
Not Hispanic	1,752 (85%)	1,679 (85.2%)	64 (80%)	
Other/unknown	18 (0.9%)	18 (0.9%)	0 (0%)	
Multiparous	1,514 (73.6%)	1,440 (73.2%)	65 (81.2%)	0.142
Gestational age (weeks)	38.4 (2.5)	38.4 (2.5)	38.3 (2.4)	0.645
Pregravid BMI (kg/m²)	29.1 (7.4)	29.2 (7.4)	26.0 (5.5)	0.001
Hypertension in pregnancy	391 (19%)	368 (18.7%)	18 (22.5%)	0.477
Gestational diabetes	313 (15.2%)	297 (15.1%)	13 (16.2%)	0.898
Polyhydramnios	64 (3.1%)	57 (2.9%)	6 (7.5%)	0.044

Nearly half of the cohort identified as White (n=973; 47.2%), and most patients were multiparous (n=1,514; 73.6%). Cesarean deliveries were scheduled in 904 cases (43.9%) and were predominantly performed via low-transverse incision (n=2,013; 97.7%) under spinal anesthesia (n=1,346; 65.3%). Delivery characteristics and postpartum clinical outcomes stratified by postoperative hemoglobin level are presented in Table 2.

**Table 2:** Delivery characteristics and postoperative clinical outcomes stratified by postoperative hemoglobin level Delivery characteristics and postoperative clinical outcomes among patients undergoing cesarean delivery stratified by postoperative hemoglobin level. Continuous variables are presented as mean (standard deviation), and categorical variables are presented as number (percentage). P-values were calculated using Student's t-test for continuous variables and chi-squared or Fisher's exact tests for categorical variables as appropriate.

	Overall (N=2,061)	Hemoglobin ≥7 g/dL (N=1,970)	Hemoglobin <7 g/dL (N=80)	P-value
Delivery type				
Low transverse	2,013 (97.7%)	1,924 (97.7%)	78 (97.5%)	1
Other	48 (2.3%)	46 (2.3%)	2 (2.5%)	
Preoperative hemoglobin (g/dL)	11.7 (1.3)	11.8 (1.2)	10.3 (1.3)	<0.001
Postoperative hemoglobin (g/dL)	9.4 (1.4)	9.5 (1.3)	6.4 (0.5)	<0.001
IV magnesium at delivery	192 (9.3%)	182 (9.2%)	10 (12.5%)	0.393
Additional uterotonics administered	271 (13.1%)	241 (12.2%)	28 (35%)	<0.001
Postpartum blood transfusion	116 (5.6%)	63 (3.2%)	53 (66.3%)	<0.001
Postpartum hemorrhage	358 (17.4%)	332 (16.9%)	26 (32.5%)	0.001
Placental abruption	52 (2.5%)	47 (2.4%)	5 (6.2%)	0.073
Postoperative tachycardia	449 (21.8%)	407 (20.7%)	37 (46.2%)	<0.001
Postoperative hypotension	132 (7.9%)	127 (7.9%)	3 (5%)	0.566
Postoperative oliguria	2 (0.2%)	2 (0.2%)	0 (0%)	1

Severe postpartum anemia (hemoglobin <7 g/dL) occurred in 80 patients (3.8%), while 1,970 patients (96.2%) had postoperative hemoglobin ≥7 g/dL. A total of 116 patients (5.6%) received a postpartum blood transfusion.

Patients with severe postpartum anemia had lower preoperative hemoglobin levels (10.3±1.3 g/dL vs. 11.8±1.2 g/dL; p<0.001) and were more likely to receive additional uterotonic agents (28/80, 35%) compared with patients without severe anemia (241/1,970, 12.2%) (χ²(1)=34.8; p<0.001; Cramér's V=0.13).

Postpartum hemorrhage occurred more frequently among patients with severe anemia (26/80, 32.5%) compared with those without severe anemia (332/1,970, 16.9%) (χ²(1)=11.4; p=0.001; Cramér's V=0.07).

Placental abruption was more common in the severe anemia group (5/80, 6.2%) compared with the non-severe anemia group (47/1,970, 2.4%), although this difference did not reach statistical significance (χ²(1)=3.2; p=0.073; Cramér's V=0.04).

Postoperative tachycardia occurred in 37/80 patients (46.2%) with severe anemia compared with 407/1,970 patients (20.7%) without severe anemia (χ²(1)=28.2; p<0.001; Cramér's V=0.12).

**Figure 1:**
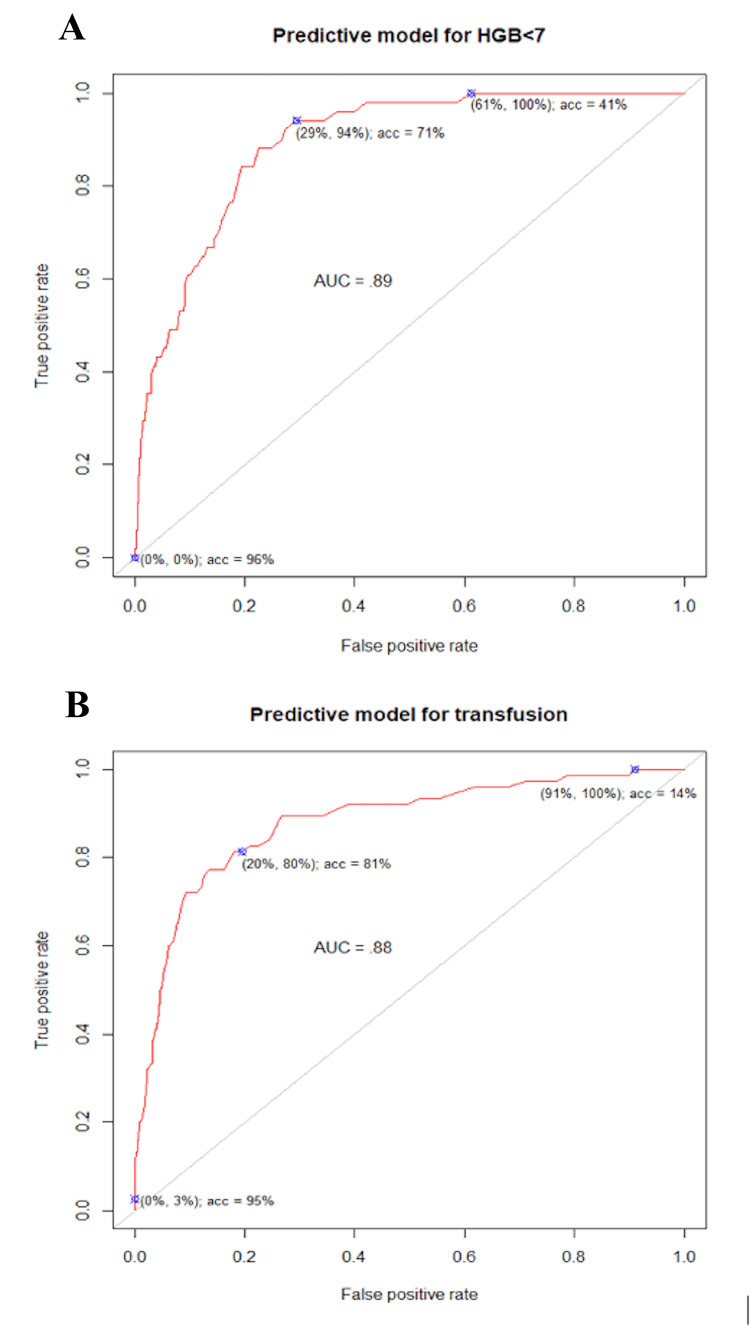
ROC curves for predictive models of severe postpartum anemia and blood transfusion following cesarean delivery (A) ROC curve for the predictive model identifying severe postpartum anemia (hemoglobin <7 g/dL). The model demonstrated strong discrimination with an AUC of 0.89. (B) ROC curve for the predictive model identifying patients requiring postpartum blood transfusion. The model demonstrated strong discrimination with an AUC of 0.88. ROC: receiver operating characteristic; AUC: area under the curve

The optimized elastic net logistic regression model (alpha=1.0, LASSO) retained two predictors of severe postpartum anemia: preoperative hemoglobin level (β=−0.55) and number of uterotonic agents administered (β=0.24). The model demonstrated strong discrimination for predicting severe anemia (AUC=0.89; Figure 1A).

Applying a prevalence-based probability threshold of 3.5%, aligned with the institutional incidence of severe anemia, reduced postoperative hemoglobin testing by approximately 65% while maintaining 90% sensitivity for identifying severe postpartum anemia. A similar model predicting postpartum blood transfusion also demonstrated strong discrimination (AUC=0.88; Figure 1B).

In exploratory analyses, linear regression modeling of continuous postoperative hemoglobin identified several variables associated with lower hemoglobin levels, including maximum postpartum heart rate, postpartum hemorrhage, ethnicity, gestational age, coagulopathy, cesarean priority, polyhydramnios, and uterine rupture (F(50,1426)=36.7; p<0.001).

## Discussion

In this large retrospective cohort, severe postpartum anemia following cesarean delivery was uncommon, occurring in only 3.8% of patients. Despite this low prevalence, routine postoperative hemoglobin testing remains common in many institutions, even though professional guidelines increasingly emphasize selective, symptom-driven evaluation rather than universal laboratory testing [6,11]. Our findings demonstrate that a simple prediction model relying solely on preoperative hemoglobin level and uterotonic use provides strong discrimination for identifying patients at risk for severe postpartum anemia. Applying this model in our cohort reduced postoperative hemoglobin testing by approximately 65% while maintaining high sensitivity, suggesting meaningful potential to optimize postpartum care and reduce unnecessary laboratory utilization.

These findings are consistent with prior literature in gynecologic surgery showing limited clinical benefit to routine postoperative hematologic testing. Chamsy et al. reported that only 0.48% of patients undergoing total laparoscopic hysterectomy required transfusion, all of whom were symptomatic [12]. Likewise, Kohli et al. observed a 1.9% transfusion rate following elective gynecologic procedures, with all transfused patients demonstrating clinical signs of anemia [13]. Although fewer studies have evaluated this question in obstetric populations, available data similarly suggest that routine postoperative hemoglobin testing after uncomplicated cesarean delivery rarely alters management and may increase healthcare costs without improving outcomes. Our findings extend this literature by providing a simple risk stratification approach that may help operationalize selective testing strategies in routine obstetric practice.

The model's reliance on preoperative hemoglobin and uterotonic use is clinically intuitive and aligns with the established predictors of postpartum anemia and hemorrhage. Predelivery anemia is a well-recognized risk factor for postpartum transfusion and severe anemia, and uterotonic administration often reflects increased concern for uterine atony or hemorrhage during the immediate postpartum period [8]. National data suggest postpartum transfusion rates of approximately 4-7%, with substantially higher rates among patients experiencing postpartum hemorrhage [2,14]. In our cohort, 5.6% of patients received transfusions, and more than half of the transfusions occurred at hemoglobin values ≥7 g/dL, underscoring that transfusion decisions frequently depend on clinical symptoms rather than absolute laboratory thresholds. These findings further support the role of clinical assessment combined with targeted laboratory evaluation rather than routine universal testing.

Importantly, current professional guidance does not recommend routine postoperative CBC testing following cesarean delivery. Instead, organizations such as the American College of Obstetricians and Gynecologists emphasize individualized postpartum assessment with laboratory testing guided by clinical findings such as hemorrhage, tachycardia, oliguria, or symptoms of anemia [15]. Despite these recommendations, many institutions continue to obtain routine postoperative hemoglobin measurements. Our findings suggest that simple clinical predictors may help bridge this gap by identifying patients in whom postoperative laboratory evaluation is most likely to provide clinically actionable information.

The strengths of this study include its large sample size, inclusive health system dataset, comprehensive variable capture, and use of a robust yet interpretable modeling approach. Because the final model relies on only two readily available clinical variables, it may be easily implemented within existing postoperative care pathways without requiring complex calculations or additional data collection. However, several limitations should be considered. The retrospective design introduces the possibility of unmeasured confounding, and the study was conducted within a single health system, which may limit generalizability. In addition, institutional practices related to postoperative hemoglobin testing, transfusion thresholds, and use of uterotonic agents may vary across settings, which could influence both model predictors and outcome ascertainment. The timing of postoperative hemoglobin measurement may also differ across institutions; because hemoglobin levels typically reach their nadir 24-48 hours after delivery, variation in the timing of laboratory assessment could affect the detection of severe anemia and model performance. External validation in diverse populations and practice environments will therefore be important to confirm model performance and generalizability.

This simplified predictive model provides a clinically practical approach to identifying patients at the highest risk for severe postpartum anemia following cesarean delivery. By helping identify patients most likely to benefit from postoperative hemoglobin assessment, this approach may support a more selective testing strategy while avoiding unnecessary laboratory evaluation in low-risk individuals. Future studies should validate this model across multiple institutions and evaluate its impact on clinical decision-making, workflow, and health system costs.

## Conclusions

This study provides a practical approach for identifying patients who are most likely to develop severe postpartum anemia after cesarean delivery. Using a small number of routinely available clinical factors, including preoperative hemoglobin level and the need for uterotonic agents, this model may help clinicians determine when postoperative hemoglobin testing is most likely to provide clinically meaningful information. In our cohort, application of this approach substantially reduced the number of postoperative laboratory tests while maintaining high sensitivity for identifying severe anemia.

These findings support a more selective, risk-based approach to postoperative laboratory evaluation following cesarean delivery. Incorporating targeted hemoglobin testing into routine postpartum care may reduce unnecessary laboratory use, improve resource utilization, and streamline postoperative management while maintaining patient safety. Future studies should focus on external validation of this model and evaluate how risk-based testing strategies may be integrated into standardized postpartum care pathways across diverse clinical settings. Given that this study was conducted within a single health system, validation in other practice environments will be important to confirm generalizability.
